# Emergence of serotype K1 *Klebsiella pneumoniae* ST23 strains co-producing the plasmid-mediated AmpC beta-lactamase DHA-1 and an extended-spectrum beta-lactamase in Korea

**DOI:** 10.1186/s13756-016-0151-2

**Published:** 2016-11-28

**Authors:** Hae Suk Cheong, Doo Ryeon Chung, Chaeyoeng Lee, So Hyun Kim, Cheol-In Kang, Kyong Ran Peck, Jae-Hoon Song

**Affiliations:** 1 Division of Infectious Diseases, Department of Internal Medicine, Konkuk University Medical Center, Konkuk University School of Medicine, Seoul, Republic of Korea; 2 Division of Infectious Diseases, Department of Internal Medicine, Samsung Medical Center, Sungkyunkwan University School of Medicine, 81 Irwon-Ro, Gangnam-gu, Seoul, 06351 Republic of Korea; 3 Asia Pacific Foundation for Infectious Diseases (APFID), Seoul, Korea

**Keywords:** *Klebsiella pneumoniae*, Extended-spectrum beta-lactamase, AmpC beta-lactamase

## Abstract

**Background:**

Serotype K1 *Klebsiella pneumoniae* has emerged as an important community pathogen causing various infections, including liver abscesses. Although serotype K1 *K. pneumoniae* community isolates have been reported as susceptible to most classes of antimicrobial agents, a few cases of infection caused by extended-spectrum beta-lactamase (ESBL)-producing serotype K1 *K. pneumoniae* have recently been reported in Asian countries. We identified three ESBL-producing strains of serotype K1 *K. pneumoniae* and conducted a molecular characterization of their drug resistance.

**Methods:**

Three ESBL-producing serotype K1 *K. pneumoniae* ST23 strains were identified from strains in the Asian Bacterial Bank. Antimicrobial susceptibility testing was performed using the broth microdilution method, and ESBL production was tested by the double-disk synergy test and a confirmatory test. PCR was performed to detect the genes for plasmid-mediated ESBL and AmpC beta-lactamases.

**Results:**

All three strains were resistant to cefotaxime, ceftazidime, and piperacillin/tazobactam, and all were determined to be ESBL-producers. No known ESBL genes, including *bla*_SHV_, *bla*_TEM_, *bla*_CTX-M_, *bla*_GES_, *bla*_PER_, and *bla*_VEB_, were detected among the three strains. Of all plasmid-mediated AmpC beta-lactamase (PAB) genes, including *bla*_DHA-1_, *bla*_CMY_, *bla*_FOX_, and *bla*_MOX_, the *bla*_DHA-1_ gene was detected in two of the strains. The PFGE patterns revealed that the two isolates carrying *bla*_DHA-1_ were closely related (84% similarity).

**Conclusions:**

No ESBL genes were detected among three ESBL-producing serotype K1 *K. pneumoniae* ST23 strains. Two strains contained the PAB gene *bla*_DHA-1_. The emergence of resistant strains of community-origin serotype K1 *K. pneumoniae* has important implications for effective treatment and infection control practices.

## Background

The rates of extended-spectrum beta-lactamase (ESBL)-production in *Klebsiella pneumoniae* are very high in hospitals worldwide, and highly resistant strains such as *K. pneumoniae* carbapenemase (KPC) or New Delhi metallo-beta-lactamase (NDM)-producing *K. pneumoniae* have been rapidly spreading between countries. Treatment of infections caused by multidrug-resistant *K. pneumoniae* has been a challenge, and there is increasing concern about the economic impact of these bacteria.

Serotype K1 *K. pneumoniae* has been reported as the predominant serotype among those isolates causing liver abscesses [1, 2]. Most K1 strains isolated from liver abscess cases belong to sequence type (ST) 23 in Asian countries [3, 4]. Although ESBL *K. pneumoniae* and KPC-producing *K. pneumoniae* have become globally widespread, a distinctive characteristic of K1 *K. pneumoniae* ST23 has been good susceptibility to most antibiotic classes (except ampicillin and piperacillin) [5], however, ESBL-producing K1 *K. pneumoniae* strains have begun to be reported recently [6, 7]. In this study, we report serotype K1 *K. pneumoniae* strains co-producing the plasmid-mediated AmpC beta-lactamase (PAB) DHA-1 and ESBL.

## Materials and methods

Three ESBL-producing serotype K1 *K. pneumoniae* ST23 strains were identified from strains in the Asian Bacterial Bank (Asia Pacific Foundation for Infectious Diseases, Seoul, Korea), which had been collected during bacteremia studies in Korea between 2006 and 2008.

In vitro antimicrobial susceptibility testing was performed using the broth microdilution method according to the Clinical and Laboratory Standards Institute (CLSI) guidelines [8]. Ten antimicrobial agents were tested, including ampicillin (AMP), amikacin (AMI), ceftazidime (CAZ), tetracycline (TET), cefepime (CPM), cefotaxime (CTX), ciprofloxacin (CIP), rifampicin (RIF), and piperacillin/tazobactam (P/T). For ESBL-positive candidates, which presented a ceftazidime or cefotaxime minimum inhibitory concentration (MIC) ≥2 mg/L, production of ESBL was confirmed by the double-disk synergy test using BBL™ Sensi-Disc™ kits (Becton Dickinson, Franklin Lakes, NJ, USA) according to CLSI guidelines [8]. Quality control for ESBL production was performed using *E. coli* ATCC 25922 and *K. pneumoniae* ATCC 700603.

Double-disk synergy test-positive isolates were further tested by polymerase chain reaction (PCR) and sequence analyses to determine the gene responsible for the ESBL phenotype in the ESBL producers. In the ESBL-producing *K. pneumoniae* isolates, *bla*_TEM_, *bla*_SHV_, *bla*_CTX-M_, *bla*_GES_, *bla*_PER_, and *bla*_VEB_ genes were tested by PCR. In addition, *bla*_DHA-1_, *bla*_CMY_, *bla*_FOX_, *bla*_MOX,_ *bla*_IMP_, *bla*_VIM_, *bla*_OXA_, and *bla*_NDM_ genes were tested. The sequences were compared with those in the GenBank nucleotide database for subtyping.

To determine serotype K1, PCR was conducted using a primer pair specific for *mag*A, which is a gene specific for the K1 antigen. The primers were chosen as previously described: forward, 5′GGTGCTCTTTACATCATTGC-3′, and reverse, 5′-GCAATGGCCATTTGCGT TAG-3′ [9]. Multilocus sequence typing (MLST) was conducted using the nucleotide sequences of seven housekeeping genes (*gagA*, *infB*, *mdh*, *pgi*, *phoE*, *rpoB*, and *tonB*) as previously described [10]. For pulsed field gel electrophoresis (PFGE), agarose-embedded bacterial genomic DNA was digested with 20 U of *Xba*I. The restriction fragments were separated by gel electrophoresis in 0.5 × Tris-borate-EDTA buffer. Electrophoresis was performed using a CHEF Mapper XA (Bio-Rad Laboratories, Hercules, CA, USA). The PFGE patterns were analyzed using GelCompar II version 6.1 (Applied Maths, Belgium).

## Results

Of the 120 *K. pneumoniae* isolates, 20 (16.7%) were ESBL-producers. Among the ESBL-producers, only three were determined to be serotype K1 (KPN1, KPN2, and KPN3). Antimicrobial susceptibilities of the three isolates are shown in Table 1. All three strains were resistant to ampicillin, piperacillin-tazobactam, cefotaxime, ceftazidime, and rifampin. Two strains (KPN2 and KPN3) were also resistant to amikacin and ciprofloxacin. One strain (KPN2) was resistant to imipenem. In all three strains, no ESBL genes, including *bla*_TEM_, *bla*_SHV_, *bla*_CTX-M_, *bla*_GES_, *bla*_PER_, and *bla*_VEB_, were detected. Among the PAB genes (*bla*_DHA-1_, *bla*_CMY_, *bla*_FOX_, and *bla*_MOX_), only *bla*_DHA-1_ was detected in two strains (KPN2 and KPN3). Carbapenemase genes were also not detected in all three isolates (Table 2). Clonal relatedness was investigated by PFGE for the two strains. Analysis of the PFGE patterns showed that the two isolates carrying *bla*_DHA-1_ were closely related (84% similarity).

**Table 1:** Antimicrobial susceptibilities in K1 *K. pneumoniae* ST23 strains

Strain	MICs and antimicrobial susceptibility																			
	AMP		P/T		CTX		CAZ		CPM		CIP		AMI		TET		IMI		RIF	
KPN1	>64	R	>256	R	16	R	>64	R	>64	R	0.125	S	4	S	1	S	0.5	S	64	R
KPN2	>64	R	>256	R	32	R	>64	R	32	R	4	R	>128	R	4	S	4	R	64	R
KPN3	>64	R	>256	R	32	R	>64	R	0.5	S	2	I	>128	R	2	S	0.125	S	64	R

*MIC* minimum inhibitory concentration, *R* resistant, *I* intermediate, *S* susceptible, *AMP* ampicillin, *P/T* piperacillin/tazobactam, *CTX* cefotaxime, *CAZ* ceftazidime, *CPM* cefepime, *CIP* ciprofloxacin, *AMI* amikacin, *TET* tetracycline, *IMI* imipenem, *RIF* rifampin

**Table 2:** Detection of ESBL genes and plasmid-mediated AmpC β-lactamase genes in K1 *K. pneumoniae* ST23 strains

Strain	ESBL genes										Plasmid-mediated AmpC β-lactamase genes						Others			
	TEM	SHV	CTX-M	PER	KPC	PER	VEB	GES/IBC	SME	IMI/NMC	CMY	ACT	FOX	DHA	ACC	MOX	IMP	VIM	OXA	NDM
KPN1	-	-^b^	-	-	-	-	-	-	-	-	-	-	-	-	-	-	-	-	-	-
KPN2	-^a^	-^b^	-	-	-	-	-	-	-	-	-	-	-	DHA-1	-	-	-	-	-	-
KPN3	-	-^b^	-	-	-	-	-	-	-	-	-	-	-	DHA-1	-	-	-	-	-	-

^a^TEM-1 (not an ESBL gene), ^b^SHV-11 (not an ESBL gene)

## Discussion

Serotype K1 *K. pneumoniae* ST23 is, a highly virulent pathogenic strain causing invasive community-acquired infections, that is widespread in its geographical distribution in Asia [5]. Fortunately, ST23 strains have shown good susceptibility to most antibiotics. However, the advent of multidrug resistance with high transmission potential in *K. pneumoniae* serotype K1 causes serious concerns. A few strains of ESBL-producing serotype K1 *K. pneumoniae* have already been reported in Asian countries [6, 7, 11]. In the present study, we report serotype K1 *K. pneumoniae* strains co-producing the PAB DHA-1 and ESBL. We found three isolates that were phenotypically ESBL producers, but we could not detect any *bla* genes responsible for the ESBL phenotype. Therefore, other ESBL genotypes may be involved. In addition, two of the three isolates were identified as PAB. Recently, gram-negative organisms that produce both ESBLs and PAB enzymes have increasingly been described worldwide [12–15]. To our knowledge, this is the first report of serotype K1 *K. pneumoniae* producing both ESBL and PAB. Both ESBLs and PAB enzymes are associated with broad, multidrug resistance because multiple antibiotic-resistance genes exist on the same plasmid [16]. One isolate (KPN3) in our study showed carbapenem resistance in the broth microdilution test. Subsequently, we used PCR analysis to determine the presence of carbapenemase genes, such as *bla*_IMP_, *bla*_VIM_, *bla*_OXA_, and *bla*_NDM_; however, none of these genes were detected. The frequency of PAB transmission may be higher than initially thought, especially if the spread of resistance mimics the trend that we have seen occurring over the past few years for ESBLs. Moreover, porin alteration, combined with the production of ESBL or PAB, has been demonstrated to confer carbapenem resistance [17, 18]. We also characterized antimicrobial susceptibility between non-serotype K1 and serotype K1 ESBL-producing *K. pneumoniae*. Although the number of total isolates was too small to draw definite conclusions, the resistant rates of piperacillin-tazobactam and imipenem in K1 isolates tended to be high. It is uncertain whether the increase of antimicrobial resistance in serotype K1 *K. pneumoniae* is connected with any one particular resistance gene.

The emergence of multidrug-resistant strains with high transmission potential in serotype K1 *K. pneumoniae* is of great concern due to limited alternative treatment options and the possibility of global dissemination. Careful surveillance of resistant strains and adequate infection prevention and control measures are necessary.

## Abbreviations

ESBL: Extended-spectrum beta-lactamase
PAB: Plasmid-mediated AmpC beta-lactamase
PFGE: Pulsed-field gel electrophoresis
KPC: *Klebsiella pneumoniae* carbapenemase
NDM: New Delhi metallo-beta-lactamase
CLSI: Clinical and Laboratory Standards Institute
MIC: Minimum inhibitory concentration
PCR: Polymerase chain reaction
